# Dopamine D4 receptor gene polymorphism (DRD4 VNTR) moderates real-world behavioural response to the food retail environment in children

**DOI:** 10.1186/s12889-021-10160-w

**Published:** 2021-02-03

**Authors:** Catherine Paquet, Andre Krumel Portella, Spencer Moore, Yu Ma, Alain Dagher, Michael J. Meaney, James L. Kennedy, Robert D. Levitan, Patricia P. Silveira, Laurette Dube

**Affiliations:** 1 Australian Centre for Precision Health, Allied Health and Human Performance, University of South Australia, Adelaide, SA Australia; 2 Faculté des Sciences de L’Administration, Université Laval, Québec, QC Canada; 3 Desautels Faculty of Management, McGill Centre for the Convergence of Health and Economics, McGill University, Montreal, QC Canada; 4 Department of Psychiatry, Faculty of Medicine and Ludmer Centre for Neuroinformatics and Mental Health, Douglas Hospital Research Centre, McGill University, 6875 Boulevard Lasalle, Montréal, QC H4H 1R3 Canada; 5 Arnold School of Public Health, University of South Carolina, Columbia, SC USA; 6 Department of Neuroscience, Faculty of Medicine, McGill University, Montreal Neurological Institute, Montreal, QC Canada; 7 Psychiatric Neurogenetics Section, Centre for Addiction and Mental Health, Toronto, Canada; 8 Centre for Addiction and Mental Health, Department of Psychiatry, University of Toronto, Toronto, Canada

**Keywords:** Genetics, Obesity, Dopamine, Food environment, Food behaviour

## Abstract

**Background:**

Evidence for the impact of the food retailing environment on food-related and obesity outcomes remains equivocal, but only a few studies have attempted to identify sub-populations for whom this relationship might be stronger than others. Genetic polymorphisms related to dopamine signalling have been associated with differences in responses to rewards such as food and may be candidate markers to identify such sub-populations. This study sought to investigate whether genetic variation of the dopamine D4 receptor gene (DRD4 exon III 48 bp VNTR polymorphism) moderated the association between local exposure to food retailers on BMI and diet in a sample of 4 to12-year-old children.

**Methods:**

Data collected from a birth cohort and a community cross-sectional study conducted in Montreal, Canada, were combined to provide DRD4 VNTR polymorphism data in terms of presence of the 7-repeat allele (DRD4-7R) for 322 children aged between 4 and 12 (M (SD): 6.8(2.8) y). Outcomes were Body Mass Index (BMI) for age and energy density derived from a Food Frequency Questionnaire. Food environment was expressed as the proportion of local food retailers classified as healthful within 3 km of participants’ residence. Linear regression models adjusted for age, sex, income, cohort, and geographic clustering were used to test gene*environment interactions.

**Results:**

A significant gene*food environment interaction was found for energy density with results indicating that DRD4-7R carriers had more energy dense diets than non-carriers, with this effect being more pronounced in children living in areas with proportionally more unhealthy food retailers. No evidence of main or interactive effects of DRD4 VNTR and food environment was found for BMI.

**Conclusions:**

Results of the present study suggest that a genetic marker related to dopamine pathways can identify children with potentially greater responsiveness to unhealthy local food environment. Future studies should investigate additional elements of the food environment and test whether results hold across different populations.

**Supplementary Information:**

The online version contains supplementary material available at 10.1186/s12889-021-10160-w.

## Background

Major global shifts in the quantity and quality of food available through our food supply have been linked to the increasing trends in obesity worldwide [1]. Variations in food supply at the neighbourhood level and their link to disparities in nutrition and nutrition-related outcomes have also been extensively investigated. The evidence emerging from this body of work has however failed to provide strong evidence supporting the impact of the neighbourhood food environment on food behaviour and related cardio-metabolic risk factors, with many studies reporting null or counter-intuitive findings [2–4]. Although the equivocal evidence could be explained by methodological limitations and heterogeneity in studies [2, 5–7], results could also reflect the fact that the local food environment does influence *some* people, but not all. This idea is consistent with calls for moving away from dichotomous understanding of neighbourhood effects in terms of whether or not they do matter to understanding of *how* and *for whom* they matter [8, 9].

While neuroscience studies have assessed how individuals respond to various food cues in controlled experimental settings [10–12], such work has been sparsely conducted in naturalistic settings. Exceptions include a study in which food consumption in children with a greater predisposition to external eating (eating in response to external cues) was found to be more strongly related to exposure to vegetable and soft drink displays compared to children with lower external eating [13]. Another example is a study showing that neighbourhood exposure to fast-food restaurants was a stronger predictor of the frequency of visits to fast food retailers in adults considered to be more reward-sensitive [14]. Another study found that an inverse association between one’s sense of mastery, a construct of perceived control over one’s circumstances, and risk for metabolic syndrome, was more pronounced for individuals living in areas with greater exposure to fast-food restaurants [15].

Further insights into individual differences in responses to food environment can be gained by looking into the neurobiology of behavioural responses to environmental cues. From a neurobehavioural perspective, environmental reactivity results from the interaction between the characteristics or traits of individuals and the valence and strength of the environmental stimulus they are exposed to [16]. With respect to neurobiological traits, main candidates include genetic and brain markers of the dopamine neurotransmitter systems, which is responsible for modulating the functioning of neural circuitry at any time, and in any environmental context, with complementary role played by the serotonin system [17]. Dopaminergic neurons are especially important at encoding motivational value and supporting brain networks for orienting, cognition, and general motivation [17]. It is recognised that the dopamine system plays an important role in the rewarding processes underlying food intake [18, 19] and if compromised, can lead to food addiction and obesity [20]. Polymorphisms in genes that significantly affect dopamine neurotransmission are therefore potential candidate genetic markers for food environment responsiveness. In particular, the dopamine D4 receptor gene (DRD4) functionally produces inhibitory effects, and is expressed in brain regions related to planning and regulating executive functions and rewards [21]. The most frequently studied D4 polymorphisms is the 48-bp variable number of tandem repeats (VNTR) on the exon 3 of the DRD4 gene, which is repeated from 2 to 11 times, with the 7-repeat allele being associated with altered function of the receptor relative to the more common 4-repeat allele [22]. The 7-repeat (7R) allele is associated with a lower affinity for dopamine and thus, reduced inhibitory effects on postsynaptic neurons [23–25], leading to increased sensitivity to both aversive and rewarding cues. Children who are carriers of this allele have also been found to have less healthy food consumption patterns compared to non-carriers [22, 26].

The vulnerability to adverse or unhealthy environments described above, also known as the diathesis-stress view, represents only one side of variation in responsiveness to environment. It has been suggested that differences in dopaminergic pathway could lead individuals to respond more intensely to not only adverse (e.g. obesogenic) conditions, but also supportive (health-promoting) environments [27], translating into the well-established “for-better–and-for-worse” pattern of susceptibility [28]. This hypothesis known as the *differential susceptibility hypothesis* [16, 29] suggests that individual variations in the magnitude of biological responses regulate openness or susceptibility to environmental influences, ranging from particularly harmful to favourable environments [29].

The DRD4-7R VNTR occupies a central place among genetic polymorphisms associated with differential susceptibility and other forms of behavioural plasticity [27]. For instance, evidence of differential susceptibility has been reported for cognitive and socio-emotional child development outcomes of family conditions [21], sensitivity to depression and associated intervention [30, 31], as well as sensitivity to cultural norms [32, 33]. As early evidence supporting the possibility of differential susceptibility in the domain of obesity and other diet-related health problems, Silveira and colleagues [34] found that children who were carriers of the DRD4-7R VNTR and raised under adverse low-socioeconomic status (SES), showed a stronger preference for fat compared to the control non-carriers group. On the other hand, DRD4-7R VNTR carriers raised in high-SES conditions reported lower preference for fatty foods compared to the non-carriers, supporting a “for better and for worse” pattern. A recent study also provided evidence for differential susceptibility to emotional eating in response to a positive postnatal environment according to a predicted gene expression of the prefrontal DRD4 gene [35].

Although socio-economic conditions and disadvantage have been linked to obesogenic qualities of the food environment [36], the above studies did not investigate directly the food environment. The present study was designed to explicitly investigate whether the association between a measure of adverse (obesogenic) vs. healthy food environment and dietary behaviour and obesity vary by DRD4-7R VNTR status in children and if so, whether differences in pattern of responsiveness are consistent with the diathesis-stress or the differential susceptibility hypotheses.

## Methods

Data for this study were obtained from two studies. The first study is the Brain-to-Society (BtS) diagnostic research project, a cross-sectional study conducted in 2013 examining a range of individual, household, and environmental risk factors for childhood obesity in 6–12 year children living in Montreal, Canada. The second study is the Maternal Adversity, Vulnerability and Neurodevelopment (MAVAN) birth cohort which recruited pregnant women from obstetric clinics in hospitals located in Montréal, Québec and Hamilton, Ontario from 2003 onwards, with the present study only using data from the Montreal cohort and assessments made when children were 4 years old.

### BtS recruitment and data collection

Recruitment for the BtS study was conducted by an independent research firm from an existing database of households known to (1) live in the Montreal Metropolitan area, (2) likely have children in the target age group (6-12y), and (3) have expressed a willingness to participate in academic research. From this database, 4947 households were randomly selected and contacted by phone. Contacted individuals asked about their willingness to complete a survey about children’s eating and lifestyle habits. Eligibility was confirmed eligibility for 1149 individuals, of those 616 completed the interviews. The response rate was estimated at 23% using a formula that allows for the potential eligibility of a proportion of respondents with unknown eligibility. Each participating household answered questions for one eligible child. If more than one child was eligible, the child with the next birthday was selected. Questions regarding the selected child were answered by parent/guardian with the best knowledge of the child’s daily habits. Interviews were conducted between March and August 2013 and had a duration of 50 min. Verbal consent was obtained from all participants prior to starting the phone survey and a written consent for follow-up data collections. Ethical approval covering all aspects of the research was obtained from McGill University’s Institutional Research Board.

Participants who completed the interview were invited to complete a self-administered questionnaire, which included a link to a validated web-based Food-Frequency Questionnaire (FFQ) [37]. Participants received questionnaires and consent (parent) and assent (child) forms by mail and returned them upon completion using a pre-paid envelope.

The FFQ asked participants to recall their children’s food intake over the last month based on a list of 136 individual food items or food clusters covering eight food and beverages categories including dairy products, vegetables, fruits, meat and alternatives, beverages, cereal and grain products, other foods, and supplements. Participants were asked to report the frequency of consumption of each food item on a 8- or 9-point scale from “never” to “four or more times per day” using portion sizes estimated using digital photographs of the food in standardized dinnerware and utensils based on the SU. VI. MAX food atlas [38]. The Nutrition Data System for Research [39] and the Canadian Nutrient file [40, 41] were used to calculate the energy content (calories) and the nutrient intake. Height and weight were reported by parents. At the end of the initial telephone survey, parents/guardians were asked if they would participate in a genetic sub-study by providing a saliva sample from the child and/or completing a mailed (or on-line) follow-up questionnaire. The kit was mailed to the families with written instructions on how to collect the sample, and then returned using a pre-paid envelope for DNA extraction at the Centre for Addiction and Mental Health Neurogenetics Laboratory (CAMH, University of Toronto).

### MAVAN recruitment and data collection

MAVAN recruitment was conducted in obstetric clinics in hospitals where mothers were invited from mid-pregnancy. Inclusion criteria included being 18 years of age or older, and fluent in either English or French. Exclusion criteria included serious obstetric complications during the pregnancy or delivery of the child, extreme low birth weight, prematurity (≤37 weeks’ gestation), or any congenital diseases. Dyads of mothers and their offspring were assessed longitudinally at home and in the laboratory across the child’s development (3, 6, 12, 18, 24, 36, 48, 60 and 72 months); however, this study is using data collected at 48 months of age, a time point when the FFQ was administered. The project received approvals from obstetricians from study hospitals, ethics committees and university affiliates. All participants provided informed consent. For more information on MAVAN methodology and measures, see [42].

The 48-month assessment, involved a number of laboratory assessments about the children, including food-related measures and height and weight measures. Standing height was measured to the nearest 0.1 cm without shoes with a stadiometer (PE-AIM-101;Perspective Enterprises) and body weight was measured in light clothing to the nearest 0.1 kg with a digital floor scale Tanita. Mothers completed FFQs valid for the local population [43] for their child, while the child performed behavioural tasks and measurements. Mothers were asked to report their child’s intake of various foods on a typical day in the last week, with the aid of a food and measures photo album to estimate the portion size of each food [44]. Total caloric and macronutrient intake was calculated using the NutriBase software (version NB7; CyberSoft Inc). Saliva samples were collected for genotyping.

### Outcome measures

Were (1) body mass index for age and sex calculated from parent-reported or directly measured children’s height and weight, in z-scores according to WHO growth standards (WHO, 2006), which was available for 544 children across the two samples; (2) eating behaviour as indexed by energy density calculated as the ratio of the total energy intake (in kcal) to the total daily weight of food and beverages consumed (g). These measures were selected to reflect the expected behavioural and physiological impacts of food environments promoting high-fat, high-sugar food choices. In addition, given that the study focused on genetic variability in responses to palatable food, we selected energy density as our behavioural measure due to its established correlation with the palatability and rewarding value of food [45, 46].

### Genotyping

For both samples, genomic DNA was extracted using a high-capacity membrane-based column (QuickGene810, AutoGen, Inc., Holliston, MA) and was quantitated using an A260/A280 ratio with a NanoDrop spectrophotometer (ThermoScientific, Inc., Wilmington, DE) and agarose gel electrophoresis. The DRD4 VNTR polymorphism was amplified, with 0.2 μM of DRD4 forward primer 5′-GCGACTACGTGGTCTACTCG and 0.2 μM of DRD4 reverse primer 5′-AGGACCCTCATGGCCTTG [47], using the Roche GC-Rich PCR System amplification buffer (Roche Applied Science, Inc., Mannheim, Germany) and 20 ng of genomic DNA in a volume of 25 μl. A Stratagene thermocycler (Life Technologies, Inc., Grand Island, NY) was used to heat the samples at 95 °C for 3 min, then cycled 40 times at 95 °C for 20 s, 57 °C for 20 s, and 72 °C for 1 min, followed by 72 °C for 3 min. Polymerase chain reaction products were separated and visualized on a 2% agarose gel (type 1-A, Sigma, St. Louis, MO) stained with ethidium bromide (Lichter, Barr et al. 1993). DNA genotyping was performed blind to the children’s behaviour and phenotype.

### Characterization of individually unique food environment

Individual local food retailing environment was measured using the modified Retail Food Environment Index (mRFEI)), which represents the *proportion* of local food retailers classified as healthful and was originally developed by the Centers for Disease Control and Prevention (CDC) [48]. We selected a relative measure of the retail food environment index over an absolute measure of availability of specific types of food stores in light of evidence that such indices are more robustly related to health outcomes than absolute availability measures [2]. Local food retailers included were supermarkets, grocery stores, fruit and vegetable stores, supercentres, convenience stores and fast-food restaurants located within 3 km of participants’ residence. As per previous the CDC definition [48], the following retailers were categorised as providing healthy food: supermarkets, grocery stores, fruit and vegetable stores, supercentres. The mRFEI was computed as the number of healthy food retailers over the total number of local food retailers using kernel density estimates allowing weighing retailers based on their proximity to the participant’s residence.

### Analyses

The Hardy-Weinberg equilibrium of the DRD4-7R variant was assessed using a Chi-square test with one degree of freedom. Generalized linear models accounting for spatial clustering by Census Tract through the use of Generalised Estimating Equations (GEE) estimation were used to separately model each outcome measured as a function of mRFEI, genotype, and their interaction. Analyses were conducted on the maximal available sample. Models were estimated using the GENMOD Procedure in SAS 9.4 (SAS Institute Inc., Cary, NC, USA). All analyses were adjusted for children’s age (in years), cohort (BtS or MAVAN), sex, and household income above or below a low-income cut-off. Low income cut-offs used in the BtS study was 45,000 CAD, which was the closest cut-off value to the low-income cut-offs (before tax) for 4-person households in large communities (> 500,000 population) when the data were collected in 2013 (44,340$). For MAVAN, low income cut-offs were based on Statistics Canada Low-Income Cut-offs Index (LICO) [49]. Ethnicity was not available in the BtS sample and was therefore not included in analyses. Statistical significance was set at alpha = 0.05. Probing and testing of measures to dissociate differential susceptibility from diathesis-stress were informed by approaches proposed by Roisman and colleagues [50] and Widaman and colleagues [51] and conducted using an online calculator [52]. These measures are described in Additional file 1.

## Results

### Sample description

Genetic information was available for 322 participants, of those 305 had food environment data and 300 also had household income information. Of those, BMI and energy density data were available for 279 and 230 participants, respectively. Descriptive characteristics for participants included in the BMI analytical sample, from both cohorts are presented in Table 1. The distribution of the DRD4-7R VNTR allele was in Hardy-Weinberg equilibrium (Chi-square = 0.0006, *p* = 0.98) in the sample. Both samples were similar in terms of sex representation, genotype, outcomes, and food environment measure. MAVAN cohort participants were younger, as expected, and had a higher prevalence of low income. No differences were found in mRFEI based on DRD4 7R VNTR status (*p* > 0.8).

**Table 1:** Participant characteristics included in both analytic samples by study (BtS/MAVAN) and combined

	BMI sample			Energy density sample		
	BtS (*n* = 159)	MAVAN (*n* = 120)	Combined (*n* = 279)	BtS (*n* = 135)	MAVAN (*n* = 95)	Combined (*n* = 230)
Age (years) (Mean (SD))	8.9 (1.7)	4.0 (0.0)	6.8 (2.8)	8.87 (1.73)	4.0 (0.0)	6.9 (2.7)
Sex (n(%) male)	73 (45.9%)	61 (50.8%)	134 (48.0%)	61 (45.2%)	44 (46.3%)	105 (45.6%)
DRD4-7R VNTR carriers (n(%))	55 (34.6%)	43 (35.8%)	98 (35.1%)	47 (34.8%)	34 (35.8%)	81 (35.2%)
BMI-for-age z-score^a^ (Mean (SD))	0.47 (1.77)	0.38 (0.94)	0.43 (1.47)	0.47 (1.73)	0.33 (0.88)	0.41 (1.47)
Energy density (kcal/g) ^b^ (Mean (SD))	4.60 (0.25)	4.50 (0.86)	4.56 (0.60)	4.60 (0.25)	4.52 (0.87)	4.56 (0.59)
mRFEI (Mean (SD))	23.84 (11.91)	25.85 (10.95)	24.70 (11.53)	24.19 (12.59)	24.91 (11.13)	24.49 (11.98)
Low income prevalence (n(%))	25 (15.7%)	38 (31.7)%	63 (22.6%)	20 (14.8%)	29 (30.5%)	49 (21.3%)

^a^ *n* = 215 for energy density sample; ^b^ *n* = 215 for BMI sample

### Gene*food environment results

Main and interactive effects of DRD4-7R VNTR status and the food environment index on energy density and zBMI are provided in Table 2. Results for energy density revealed main effects of DRD4-7R VNTR, with the presence of the DRD4-7R VNTR being associated with greater energy density. No main effect of the food environment was found for either outcome. A statistically significant interaction effect was found between genotyping and the mRFEI measure on energy density, which suggested that there was an inverse association between mRFEI and energy density for DRD4-7R VNTR carriers only. Results for zBMI suggest no significant main or interactive effects of DRD4-7R VNTR and food environment measures.

**Table 2:** Results of DRD4-7R VNTR *Food environment interaction for BtS-MAVAN Combined (models adjusted for age (in years), sex, low household income status (< 45 K), and geographic clustering by Census tract)

Predictors	BMI (*n* = 279)	Energy density (*n* = 230)
DRD4-7R VNTR carrier	0.056 (95%CI: −0.685, 0.797); *P* = 0.88	0.51 (95%CI: 0.199, 0.813); *P* = 0.001
mRFEI	0.006 (95%CI: −0.009, 0.021); *P* = 0.44	0.004 (95%CI: −0.002, 0.010); *P* = 0.20
mRFEI*DRD4 7R VNTR	0.006 (95%CI: −0.020, 0.031); *P* = 0.65	−0.014 (95%CI: − 0.024, − 0.004); *P* = 0.007

We explored the nature of the interaction found between mRFEI and DRD4-7R VNTR for energy density using probing and measures allowing to assess whether the interaction was consistent with the differential susceptibility or diathesis-stress hypothesis as described elsewhere [50, 51] and in Additional file 1. To do so, we first tested the assumption of linearity of relationships as suggested by Widaman and colleagues [51] by testing for quadratic and cubic terms for centred values of mRFEI within each DRD4-7R VNTR status group. None of the non-linear terms were statistically significant (P’s > 0.5). The cross-over point, that is, the value of the mRFEI where the regression lines for DRD4-7R VNTR carriers and non-carriers cross, was computed and the proportion of the sample with values of mRFEI above the cross-over point was estimated at 14.5%, suggesting that the cross-over point occurred at the higher end (i.e. healthier end) of the mRFEI range observed. This measure, known as the Proportion Affected (PA) index represents an estimate of the proportion of the population that is differentially affected by the moderator (DRD4 7R VNTR), with values closer to 0.5 considered to provide support for the differential susceptibility hypothesis whereas values closer to 0 considered to be more consistent with the diathesis-stress hypothesis (Roisman, Newman [50]).

**Fig. 1:**
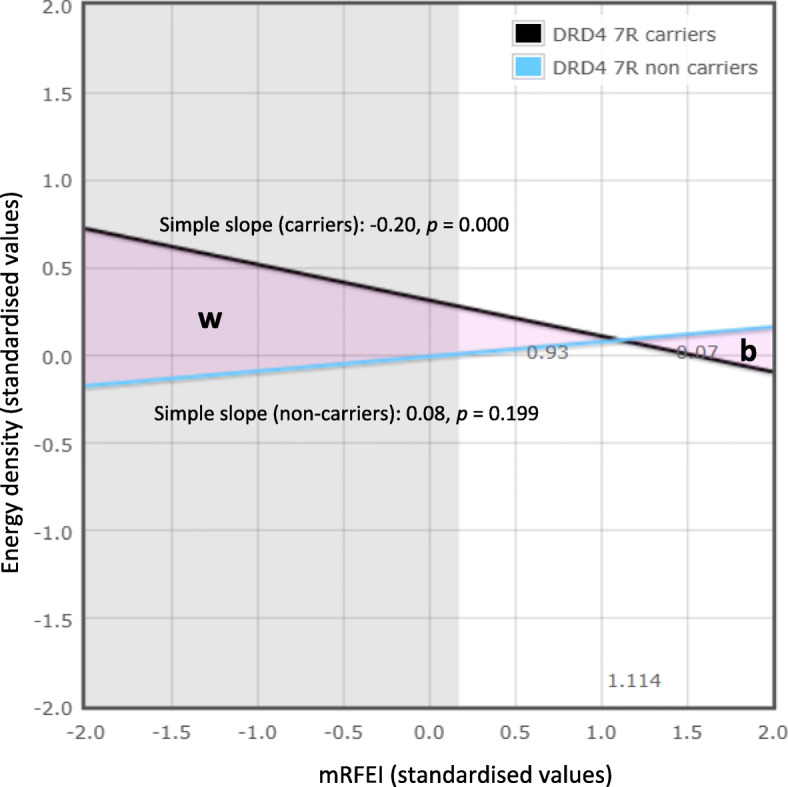
Associations between mRFEI and energy density for DRD4-7R VNTR carriers and non-carriers. Shaded area represents the area for which energy density differed between carriers and non-carriers, ‘w’ and ‘b’ indicates areas where carriers are respectively worse or better than non-carriers

The cross-over point at high values of mRFEI was confirmed by plotting and probing the relationship between mRFEI and energy density for DRD4-7R VNTR carriers and non-carriers (Fig. 1). This analysis also confirmed that an inverse association between mRFEI and energy density was only present for DRD4-7R VNTR carriers, as shown in the figure. The figure also illustrates in grey the values of mRFEI for which there was a statistically significant difference in energy density according to DRD4-7R VNTR status, which suggests that differences were only present for relatively less healthy food environments, with differences in energy density between carriers and non-carriers being approximately 0.3 SD (approximately 0.2 kcal/g) for children living in areas of average mRFEI value and 0.6 SD (0.4 kcal/g) for those in areas 1 SD below the mean (i.e. more unhealthy than average). Finally, the figure also highlights that the area for which carriers are more negatively affected by the environment than non-carriers (labelled as ‘w’ for worse) is greater than the area for which they are advantaged by the environment (‘b’ for better). Overall, the results appear to be more consistent with the diathesis-stress hypothesis than the differential susceptibility hypothesis.

## Discussion

Our data provide evidence of individual genetic differences in the behavioural impact of the commercial food environment, supporting the moderating effect of the DRD4-7R VNTR polymorphism. While results revealed that DRD4-7R carriers had overall more energy dense diets than non-carriers, this effect was more pronounced for children living in areas with proportionally more unhealthy food retailers. Further investigation of the pattern of interaction suggested that results were consistent with a diathesis-stress pattern, with no evidence of children who were carriers of the DRD4-7R VNTR polymorphism being more positively affected by healthy food environments than non-carriers, as would be expected under the differential susceptibility pattern. Moreover, evidence of association between the food retail environment and behaviour was only found for DRD4-7R VNTR carriers.

The lack of evidence for differential susceptibility contrasts with other studies conducted investigating the DRD4-7R VNTR polymorphism. In particular, a study by Silveira and colleagues [34] conducted in the MAVAN sample provided evidence of differential susceptibility to socio-economic conditions in relation to food-related outcomes, although the focus on fat preference rather than total energy density intake may explain the differences considering that variations in the dopamine signalling may be particularly and specifically linked to variations in the intake of palatable foods [26]. Another reason for the observed differences might be that the benefits associated with favourable socio-economic conditions may not depend on more healthful food sources, but perhaps more on factors such as better food education and parental support.

In contrast to the energy density results, no main or interactive effects of gene and food environment were found for zBMI. This null finding, however, does not invalidate the results for energy density as they can be due to a number of reasons. For example, dopamine pathways and DRD4 polymorphisms have also been related to physical activity levels in animals and humans [53], which may counteract the metabolic effect of a high-energy diet. Unfortunately, physical activity data were not available to test this explanation. Another potential reason could be the relatively young age of the sample, in which the cumulative impact of energy density on BMI may not have yet taken place. Finally, obesity and its associated markers are influenced by a range of inter-related social, economic, cultural and community factors. Future studies should be undertaken in larger samples while considering a range of obesogenic factors to explore potential mechanisms.

The results need to be interpreted in the context of limitations tied to naturalistic observation and small sample size of the study and they await further replication in both field and laboratory research studies. Statistical power also did not allow testing for sex differences in results, with previous studies indicating sex-specific effects [34]. In addition, our sample combined data from children from pre-school age up to 12 years old, which are likely to differ in their food preferences and level of independence with respect to food decisions. Children within this age range could also be considered to have a limited impact on household food purchasing decisions. However, children have been shown to influence supermarket purchases [54, 55]. Moreover, a study provided evidence that younger children are more likely to ask for advertised food products compared to adolescents [56]. Unfortunately, our sample size did not allow to test whether results differed by age groups. Even though the two data sources sampled children from the same metropolitan region and used the same genetic and environmental measures, the studies differed in their sampling frames, anthropometric measurement methods and food-frequency questionnaires used, which may have introduced additional sources of heterogeneity in the combined data. Ethnicity and education measures were not available in the BtS sample and were therefore not considered in the analyses. Future studies should therefore explore potential age- and ethnicity-related differences and adjust for parental education. In addition, information was not available on the genotype, phenotype or diet of the parents who play an important role in shaping the family food environment, with recent findings suggesting less healthful home food environment in mothers with lower levels of executive functioning (also related to the dopamine system) [57]. Future research is also needed to characterize the neurobiological, behavioural, and psychological endophenotypes associated with the function of the dopamine pathways through which DRD4-7R VNTR susceptibility impacts behaviour (e.g., motivated attention, reward seeking, inhibitory control, and mental flexibility) and using genome-wide approaches. There is a need to trace the multiscale pathways linking genes and environmental factors to outcomes through specific component brain processes, characterizing the neuromodulator foundation of these genetic variables in terms of the basic nature of their reactivity to environmental contexts. Finally, the food environment exposure only considered the type of food outlets available locally and not the variation in foods and marketing strategies used within each outlet and we did not have information on where participants shopped and what they bought. Future studies should consider using sales receipts, sales or loyalty programs data combined with genetic data for a more accurate portrait of purchasing decisions and their genetic and environmental influences.

## Conclusion

Results of the present study suggest that a common genetic marker related to dopamine pathways can identify children with greater responsiveness to local food environment. The results confirm the need for food environment research to move beyond determining whether food environment matters, and to investigate for whom it matters and under what conditions in order to develop the best possible strategies to those most likely to benefit from them.

## Supplementary Information

**Additional file 1.** Differential Susceptibility testing – Overview. This additional file provides additional details on the differential susceptibility testing measures used in the manuscript and their interpretation.

## Data Availability

The datasets used and/or analyzed during the current study are available from the corresponding author on reasonable request.

## Abbreviations

DRD4-7R VNTR: 7-repeat (7R) allele of the 48-bp variable number of tandem repeats (VNTR) on the exon 3 of the D4 receptor (DRD4) gene
zBMI: Body Mass Index for age expressed in z-scores according to WHO growth standards
MAVAN: Maternal Adversity, Vulnerability and Neurodevelopment (MAVAN) birth cohort
mRFEI: modified Retail Food Environment Index
BtS: Brain-to-Society (BtS) study
