# Supplementary material for: Dopamine D4 receptor gene polymorphism (DRD4 VNTR) moderates real-world behavioural response to the food retail environment in children
Source: BMC Public Health. 2021 Feb 3;21:145. doi: 10.1186/s12889-021-10160-w (PMC7856809; doi:10.1186/s12889-021-10160-w)
Supplement: Supplementary file 1 — Additional file 1. Differential Susceptibility testing – Overview. This additional file provides additional details on the differential susceptibility testing measures used in the manuscript and their interpretation. [file 12889_2021_10160_MOESM1_ESM.docx]

**Supplementary file 1**

Differential Susceptibility testing – Overview

This document provides information on the approach followed for probing significant interactions and the measures used to differentiate whether the interaction found was consistent with a **diathesis-stress pattern,** where one group is consistently more negatively affected by the environment than the other (shown on the left below), from a **differential susceptibility pattern**, where one group has better outcomes in supportive environment but worse outcomes in adverse environments (shown on the right below).


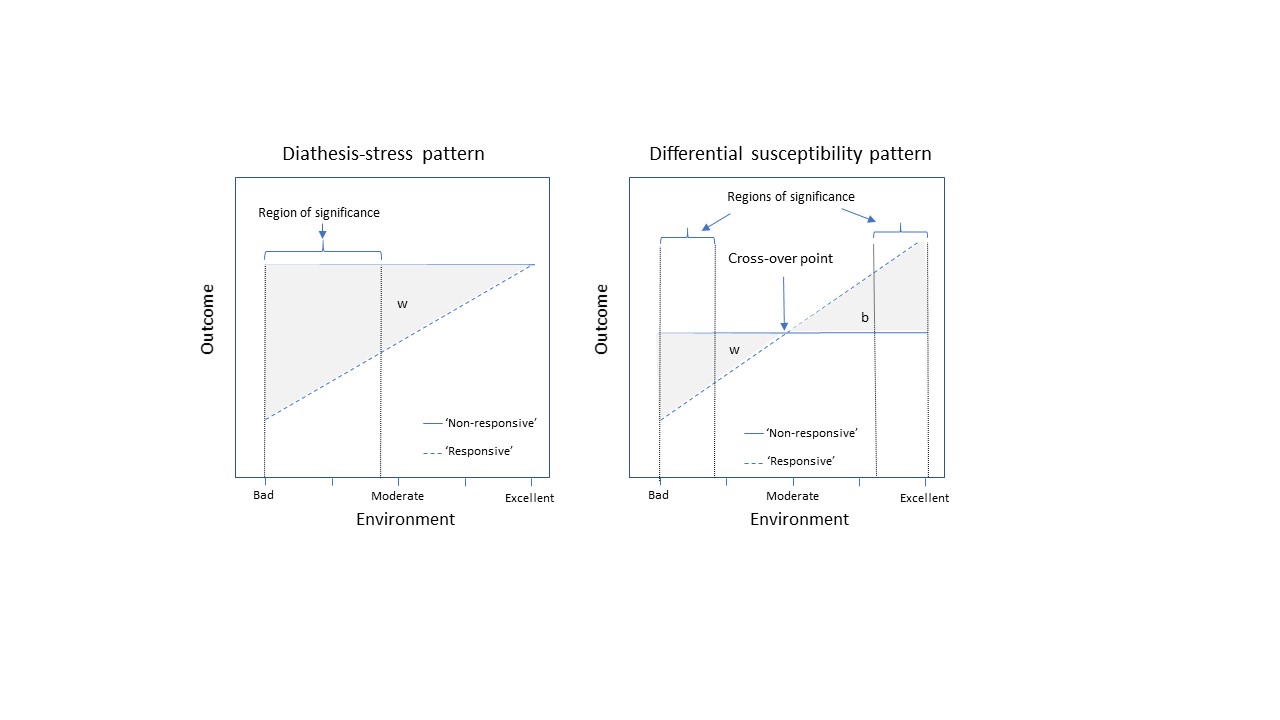


Methods have been proposed by Roisman and colleagues (1) and Widaman and colleagues (2) to compute measures illustrated in the figure above to help determine whether results of Gene*Environment interactions are consistent with the diathesis-stress vs. differential susceptibility patterns. These measures and the values supporting either the diathesis-stress or differential susceptibility pattern are described in the table below.

| Measure | Definition | Values supporting | | |
| --- | --- | --- | --- | --- |
|  |  | Diathesis Stress | Differential Susceptibility |  |
| Cross-over point | Value of X (environment) where the two lines intersect. | Cross-over point at higher end of normative range (+/- 2 SD) of X. | Cross-over point around mid-point of normative range of X |  |
| Proportion affected. | Proportion of the population that is differentially affected by the moderator (Proportion of sample with values of X above cross-over point). | Values close to 0%. | Values close to 50%. Roisman et al (1) suggest that values below 16% should lead researchers to question whether their data are consistent with the differential susceptibility pattern. |  |
| w | Region where the ‘responsive’ group has greater vulnerability (i.e. worse outcome) to environmental exposure than the ‘non-responsive’ group. | PoI values  close to 0.00  (i.e. w represents 100% of interaction region). | PoI values  close to 0.50. |  |
| b | Region where the ‘responsive’ group has better outcomes than the ‘non-responsive’ group. |  |  |  |
| Proportion of interaction (PoI) | Proportion of (b/(b+w)) |  |  |  |
| Region of significance (RoS) | The values of X for which the two groups (defined by the moderator) have statistically different outcomes | RoS at the low but not high end  of X within the normative range (+/- 2 SD) | RoS at the low and high end  of X within the normative range (+/- 2 SD) |  |

Computation

The above measures can be derived using freely available online calculators, such as the one available at <http://www.yourpersonality.net/interaction/> , which use as input regression and (co)variance estimates obtained from regression models with interaction terms where continuous predictors have been standardised prior to analysis.

**References**

1. Roisman GI, Newman DA, Fraley RC, Haltigan JD, Groh AM, Haydon KC. Distinguishing differential susceptibility from diathesis–stress: Recommendations for evaluating interaction effects. Development and psychopathology. 2012;24(2):389-409.

2. Widaman KF, Helm JL, Castro-Schilo L, Pluess M, Stallings MC, Belsky J. Distinguishing ordinal and disordinal interactions. Psychological Methods. 2012;17(4):615.
